# Comparison of the Efficacy of Intramammary or Injectable Antibiotic Administration against Staphylococcal Mastitis in Ewes

**DOI:** 10.3390/pathogens11101164

**Published:** 2022-10-09

**Authors:** Natalia G. C. Vasileiou, George C. Fthenakis, Vasia S. Mavrogianni

**Affiliations:** 1 Faculty of Animal Science, University of Thessaly, 41110 Larissa, Greece; 2 Veterinary Faculty, University of Thessaly, 43100 Karditsa, Greece

**Keywords:** control, mammary infection, mastitis, sheep, *Staphylococcus*, treatment

## Abstract

The objectives of the work were (a) to compare the efficacy of two routes for antibiotic administration in the treatment of mastitis in ewes and (b) to assess the potential importance of the timing of the initiation of the therapeutic regime on the outcome of the treatment. The ewes were allocated at random into three equal groups; intramammary inoculation with a *Staphylococcus simulans* isolate was performed, and clinical mastitis developed. The ewes in groups T1 (*n* = 6) and T2 (*n* = 6) were treated by the intramammary administration of ampicillin and dicloxacillin (two administrations with a 12-h interval). The ewes in group T3 (*n* = 6) were treated by the intramuscular injection of ampicillin and dicloxacillin (0.75 mL per 10 kg bodyweight, three injections with a 24-h interval). In the ewes in groups T1 and T3, treatment started immediately when the clinical signs of mastitis were first detected during the periodic examination of the ewes; in the ewes in group T2, treatment started 24 h after the clinical signs of mastitis were first detected. The animals were monitored clinically; mammary secretion samples were collected for bacteriological and cytological examinations. The median duration of the clinical signs was 4.75, 7.13, and 4.75 d for T1, T2, and T3; significant differences in clinical severity between the groups were seen until the 7th day post-treatment. The median duration of bacterial recovery was 3.25, 8.00, and 8.00 d for T1, T2, and T3; significant differences in the frequency of bacterial recovery between the groups were seen until (64.1%, 94.9%, and 96.2% of the samples) and after (2.9%, 16.7%, and 11.8%) the 7th day post-treatment. The median period required for the complete cure (clinical, bacteriological, and cytological) was shorter in the T1 than in the T2 and T3 ewe groups: 20.0, 32.0, and 24.5 d, respectively. The findings cover a gap in the available literature regarding the treatment of clinical mastitis in ewes. Early treatment resulted in the improved cure of the infection. The comparison of the intramammary and injectable routes for antibiotic administration indicated some benefit for the former, primarily in the post-treatment somatic cell counts.

## 1. Introduction

The importance of mastitis has been highlighted in many studies performed around the world. The infection has well-document adverse effects on the health and welfare of animals. It can also cause devastating effects on the finances of ruminant farms.

A recent detailed assessment of the international literature on mastitis in sheep has concluded that there is a paucity of information regarding the treatment and control of mastitis in that species. Specifically, of the 580 original papers on the general topic of mastitis in sheep, only 50 (8.6%) reported work on the treatment of the infection [1]. Relevant papers described treatment regimes with the administration of antibiotics in clinical cases [2,3,4] or in experimental work [5] on ovine mastitis, whilst other papers focused on supportive treatment, e.g., with non-steroid anti-inflammatory drugs [6]. Papers on the antibiotic susceptibility of bacterial isolates recovered from cases of mastitis have also been published [7,8,9], as this is important for the implementation of the correct treatment regimes. Furthermore, the determinants for the treatments (i.e., factors taken into account for decisions regarding the therapeutic strategies) followed in cases of mastitis have also been studied [10,11].

Effective therapeutic regimes against mastitis are important to restore the health and welfare of affected animals. Moreover, effective treatment is also important in control programs, as it minimizes bacterial dissemination in the environment and reduces the possibilities for infection of other animals on the farm [12]. Treatment of the infection requires the administration of antibiotics [10]. Most frequently, oxytetracycline, penicillin, and streptomycin were found to have been administered [11]. However, the small number of products licensed specifically for use in sheep leads to high rates of extra-label use of antibiotic products [10]. This lack of licensed products underlines the necessity for scientific studies investigating protocols for the treatment of the infection.

Various antibiotics have been assessed for potential administration for the treatment of ovine mastitis. For example, Naccari et al. [3] studied the efficacy of a single injection of tilmicosin (dose rate: 10 mg kg^−1^ bodyweight) against staphylococcal mastitis and reported a full cure within 7 days after treatment. In a further study, Naccari et al. [4] reported that three injections of teicoplanin (a semi-synthetic glycopeptide antibiotic) at the dose rate of 6 mg kg^−1^ of bodyweight resulted in a complete clinical and bacteriological cure of staphylococcal mastitis within up to 30 days after administration. Thereafter, the efficacy of enrofloxacin against staphylococcal mastitis (caused by *Staphylococcus aureus* or coagulase-negative staphylococci) was evaluated by Attili et al. [5], who reported that administration at the dose rate of 6 mg kg^−^^1^ of bodyweight, twice daily for three days, showed 82% efficacy based on the results of bacteriological examinations.

There is a scope and an interest in studying the treatment of mastitis caused by staphylococci as these bacteria are the most frequent causal agents of the infection [12]. *S. aureus* and coagulase-negative species (primarily *Staphylococcus simulans*, *Staphylococcus epidermidis*, and *Staphylococcus chromogenes* [13]) have been reported as aetiological agents of clinical or subclinical mastitis, particularly in dairy sheep flocks [12].

The objectives of the present work were (a) to compare the efficacy of two routes (intramammary versus injectable) for antibiotic administration in the treatment of clinical mastitis in ewes and (b) to assess the potential importance of the timing of the initiation of the therapeutic regime (at onset of clinical signs versus 24 h after their onset) on the outcome of treatment.

## 2. Results

### 2.1. Findings before Initiation of Treatment

Before the challenge (D–4, D–1), the mammary glands of all the ewes appeared clinically normal. No bacteria were isolated from the milk samples. The somatic cell counts were below 0.75 × 10^6^ cells mL^−1^. Macrophages predominated in the smears.

All the ewes in the study developed clinical signs of mastitis in the inoculated mammary gland within 18 to 36 h after the challenge (median time post-inoculation when clinical signs were first seen: 24 h for all three groups; minimum–maximum: 18–30 h, 18–30 h, and 18–36 h for T1, T2, and T3, respectively; *p* = 0.93 between groups). At that time point, there was no significant difference between the groups in the median scores for clinical severity between the three groups: 5.5 (4–6), 5.5 (4–6), and 6.0 (4–6) for T1, T2, and T3, respectively (*p* = 0.81). The scores for the ewes in group T2 further increased by the time of the initiation of treatment: 8.0 (6–9); hence, at the initiation of treatment, the difference in the clinical scores between the three groups was significant (*p* = 0.022).

Staphylococci with colonial morphology similar to that of the challenge organism were recovered in a pure culture from the inoculated gland of all the animals at the first post-challenge sampling (6 h after the challenge) and continuously thereafter until treatment. All the isolates (*n* = 20) in which the biochemical tests were employed for full identification were identified as *Staphylococcus simulans*.

Over 98% of the cells observed in the smears made from the mammary secretion samples were neutrophils.

No clinical signs were seen in the contralateral gland of any ewe. The difference in clinical scores between the inoculated and contralateral glands (median: 0, minimum–maximum: 0–0) at the time point of the first appearance of the clinical signs was significant for all three groups (*p* = 0.0001). No bacteria were recovered from the milk samples from the contralateral glands, and the somatic cell counts therein were below 0.75 × 10^6^ cells mL^−^^1^. Macrophages predominated in the smears from these samples.

### 2.2. Clinical Findings after Treatment

**Figure 1:**
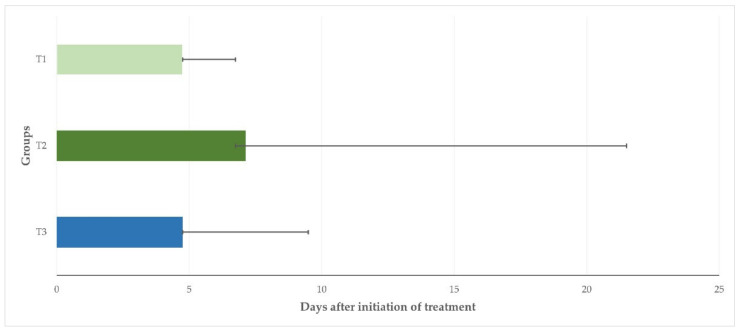
Median duration of clinical signs of mastitis in three groups of ewes after intramammary treatment performed immediately (T1) or with a 24 h delay (T2) or after systemic treatment performed immediately (T3) (bars indicate minimum–maximum).

After treatment, the clinical scores were found to be higher than on D0 for up to 1.5 (T1, T3) or 2 (T2) days. Thereafter, they progressively decreased and were found to be zero (0) in all animals in groups T1, T2, and T3 on D7, D23, and D10, respectively. The median duration of clinical signs was 4.75 d (4.75–6.75 d), 7.13 d (6.75–21.5), and 4.75 d (4.75–9.50 d) for the ewes in T1, T2 and T3, respectively (*p* = 0.026) (Figure 1).

**Figure 2:**
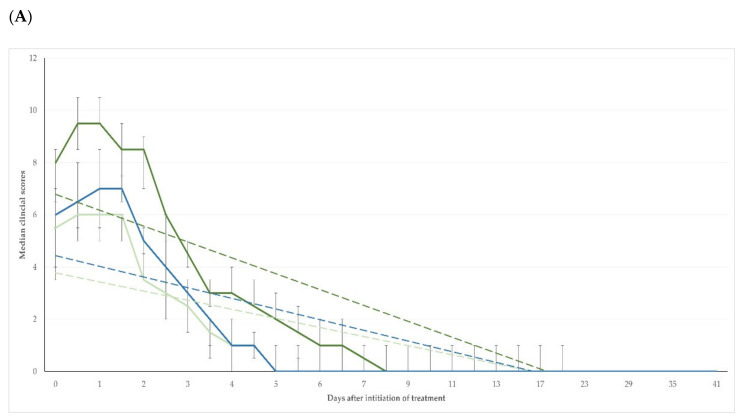

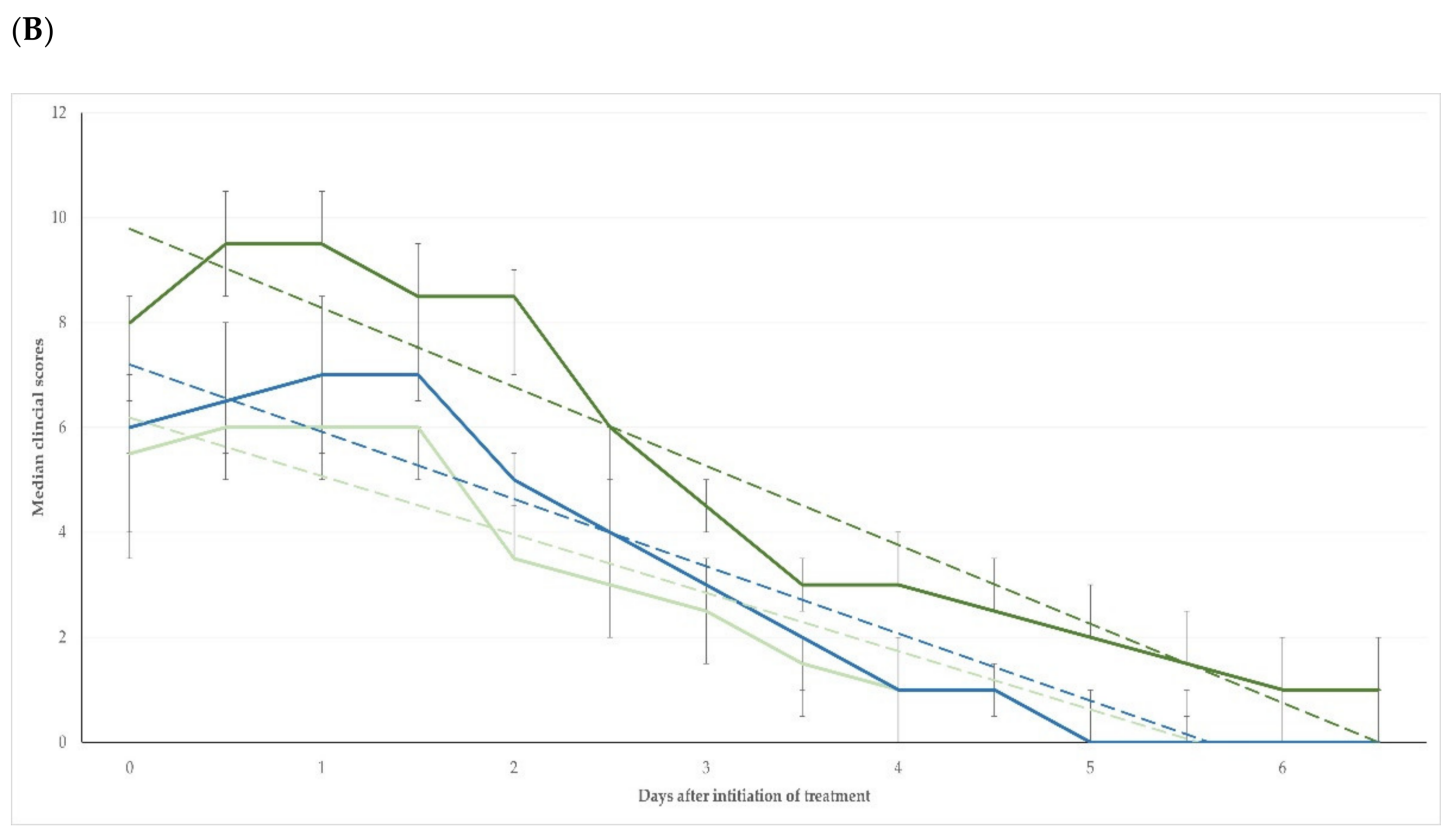
Scores for clinical severity of mastitis in three groups of ewes after intramammary treatment performed immediately (T1, light green line) or with a 24 h delay (T2, dark green line) or after systemic treatment performed immediately (T3, blue line) (dashed lines indicate respective trendlines, bars indicate minimum–maximum): (**A**) period from D0 to D41 (slopes: −0.09 ± 0.03, −0.17 ± 0.04, −0.11 ± 0.03, respectively; *p* > 0.11); (**B**) period from D0 until D7 (slopes: −1.11 ± 0.10, −1.51 ± 0.14, −1.28 ± 0.12, respectively; *p* between T1 and T2 = 0.029, for other comparisons *p* > 0.26).

The differences in clinical scores between the three groups were significant up to D6 + 12 h (*p* < 0.04); from D7 onwards, no significant differences were seen between the groups (*p* > 0.33) (Table 1 and Table S1). For the length of the study period (i.e., D0 to D41), there was no significant difference between the three groups in the changes in the clinical scores compared to the pre-treatment scores (*p* = 0.14) (Figure 2A); however, when only the period D0 to D6 + 12 h was considered, the difference in the changes in clinical scores between the three groups was significant (*p* < 0.0001) (Figure 2B).

The differences in clinical scores between the inoculated and the contralateral mammary glands were significant (*p* = 0.05) until D4 + 12 h, D6 + 12 h, and D4 + 12 h for T1, T2, and T3, respectively.

### 2.3. Laboratory Findings after Treatment

#### 2.3.1. Bacteriological Results

After treatment, staphylococci in pure culture with colonial morphology similar to that of the challenge organism, were recovered from 51 (28.3%), 91 (50.6%), and 87 (48.3%) mammary secretion samples collected from the inoculated mammary glands of ewes in groups T1, T2, and T3, respectively (*p* < 0.0001 between groups) (Table S2). In one ewe in group T1, two ewes in group T2, and two ewes in group T3 (*p* = 0.76), staphylococcal isolation was recorded intermittently, i.e., again after a temporary cessation of recovery; the median length of the cessation of isolation was 1.0 d (0.5–2.0 d). All isolates (*n* = 46) in which biochemical tests were employed for full identification, were identified as *S. simulans*.

**Figure 3:**
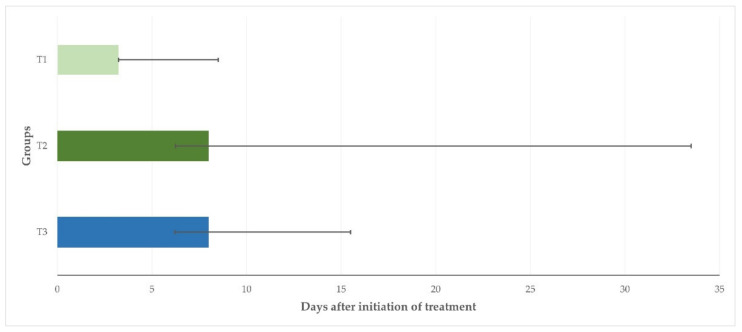
Median length of bacterial recovery from mammary secretion samples in three groups of ewes after intramammary treatment performed immediately (T1) or with a 24 h delay (T2) or after systemic treatment performed immediately (T3) (bars indicate minimum–maximum).

The median duration of bacterial recovery from the mammary secretion samples collected from the inoculated mammary glands after initiation of treatment was 3.25 d (3.25–8.50 d), 8.00 d (6.25–33.50), and 8.00 d (6.20–15.50 d) for animals in T1, T2, and T3, respectively (*p* = 0.046) (Figure 3).

Significant differences between rates of bacterial recovery were seen between the three groups for the samples collected until D7 (*p* < 0.0001; for comparison between T1 and T2, *p* < 0.0001) and from D7 to D41 (*p* = 0.005; for comparison between T1 and T2, *p* = 0.001) (Table 2).

The associations of the clinical scores with the results of the bacterial recovery from the mammary secretion samples, in accordance with the group, are shown in Table S3.

No bacteria were recovered from the milk samples obtained from the contralateral, uninoculated mammary glands of the ewes in the three groups.

#### 2.3.2. Cytological Results

After treatment, there was no difference in the proportion of samples with somatic cell counts above 0.75 × 10^6^ or 1.00 × 10^6^ cells mL^−1^ in the samples from the inoculated mammary glands of the ewes in groups T1, T2, and T3, respectively: *p* = 0.13 or *p* = 0.63, respectively (Tables S4 and S5). However, the proportion of samples with somatic cell counts above 0.50 × 10^6^ cells mL^−1^ was significantly lower among the ewes in group T1 than among the ewes in T2 and T3: 74.8%, 90.9%, and 85.2%, respectively (*p* = 0.006) (Table S5).

**Figure 4:**
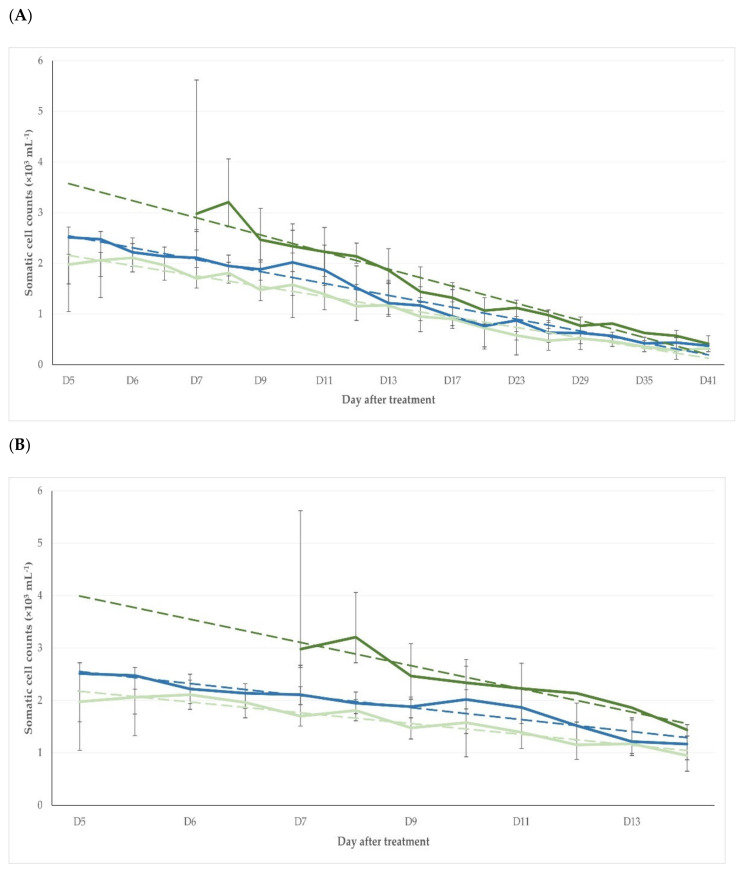
Somatic cell counts in mammary secretion samples of three groups of ewes after intramammary treatment performed immediately (T1, light green line) or with a 24 h delay (T2, dark green line) or after systemic treatment performed immediately (T3, blue line) (dashed lines indicate respective trendlines, bars indicate minimum–maximum): (**A**) period from D0 to D41 (slopes: −0.05 ± 0.01, −0.07 ± 0.01, −0.06 ± 0.01, respectively; *p* > 0.07 for all comparisons); (**B**) period from D0 until D17 (slopes: −0.12 ± 0.01, −0.22 ± 0.03, −0.13 ± 0.01, respectively; *p* between T1 and T2 and between T1 and T3 < 0.0001, for comparison between T1 and T3, *p* = 0.48).

Overall, the somatic cell counts were significantly lower in the T1 than in the T2 and T3 ewes (Table 3, Figure 4). Moreover, the somatic cell counts were over 0.50 × 10^6^ or 0.75 × 10^6^ or 1.00 × 10^6^ cells mL^−1^ for a significantly shorter duration in the T1 than in the T2 and T3 ewes in all three of the thresholds employed: *p* = 0.004, *p* = 0.004 or *p* = 0.039, respectively (Table S6).

Neutrophils predominated in the smears made from the samples from all ewes up to D5 to D5 + 12 h. Thereafter, lymphocytes were also seen in the smears, and these cells were the main cell type from D10 (group T2) or D12 to D13 (groups T1 and T3) onwards.

### 2.4. Achievement of Complete Cure

A complete cure was achieved significantly earlier in the ewes in group T1 than in the ewes in groups T2 and T3, independently of the value of somatic cell counts used for the definition of the outcome: *p* = 0.004, *p* = 0.004, or *p* = 0.033 (Table S7).

## 3. Discussion

### 3.1. Time of Initiation of Treatment

The results underline the importance of the early initiation of the treatment of mastitis, i.e., with the onset of the first clinical signs. The ewes in which treatment was delayed, required a significantly longer time for a complete cure, as confirmed by the results of the clinical, bacteriological, and cytological examinations. In cases of delayed treatment, invading bacteria would multiply within the infected mammary gland, leading to expansion of the infection and extension of the lesions within the gland. Previous studies have indicated that damage to the mammary parenchyma started within two to three days after infection; after the 4th day of infection, some mammary lesions can be irrevocable [13]. The alveolar destruction that occurs after the 4th day of infection leads to permanent damage in the mammary parenchyma and consequently to the fibrotic lesions therein [13]. Moreover, the destruction of vessels within the mammary gland [13] may potentially contribute to reducing the number of leucocytes in the mammary gland, thus hindering effective bacterial clearance.

Previous reports, based on a theoretical hypothesis, have advocated the immediate start of treatment with the first signs of mastitis [14]. The present results provide experimental corroboration of that hypothesis.

To note that the present study focused on staphylococcal mastitis and generalizations to other mastitis pathogens should be extrapolated cautiously. One may also argue that the results are of lesser significance for cases of mastitis caused by other pathogens, for example those that can be cleared quickly by the animal’s defenses, such as *Escherichia coli* [15,16]. Whilst these arguments are valid, it should also be noted that in ewes the majority of cases of mastitis are caused by staphylococci [17,18], which was the main reason for performing this study in an experimental staphylococcal mastitis model.

In the animals in which the treatment was delayed, the bacteriological clearance took significantly longer, likely as the result of higher bacterial populations therein and the consequent longer period allowed for multiplication. These higher numbers needed a longer period for clearance and also resulted in the recrudescence of shedding more often.

Hence, and in view of the present results, the early initiation of the treatment of clinical mastitis appears to be an important determinant of the favourable outcome of the procedure.

### 3.2. Intramammary versus Injectable Administration of Antibiotics

For the comparison of the two routes of administration, we included the only two pharmaceutical products licenced in Greece for veterinary use which contained the same active ingredients (albeit in different quantities) and were available for intramammary and injectable administration. This way, we avoided differences in results due to the potentially different efficacy of the active ingredients of the products, as this would have been a limitation in the comparison of the two routes of administration. To note is that the total quantity of each of the two active ingredients administered to the respective ewes was higher after treatment with the injectable form: 1286.6 mg ampicillin and 645.3 mg dicloxacillin versus 376.4 mg and 382.8 mg after treatment with the intramammary form. No further comparisons can be made between the two products regarding the quantities of active ingredients administered to the ewes.

After systemic administration, the two active ingredients of the product (i.e., dicloxacillin and ampicillin) are rapidly absorbed and distributed to the various organs and tissues of the treated animals. Following intramammary administration, a high proportion (>80%) of both active ingredients is released in the milk within the mammary gland, leading to high concentrations. In the case of both pharmaceutical products, elimination occurs in an unmodified form, principally by the urine or the milk, after systemic or intramammary administration, respectively [19,20].

When comparing the two routes, no difference was found in the recession of the clinical signs of the infection. This can possibly explain the preference for using injectable products for the treatment of clinical mastitis; in a recent study, it was reported that in 89% of sheep farms in Greece solely the injectable form was employed for the treatment of clinical mastitis [11]. In this respect, it should also be noted that the administration of the antibiotics by the injectable route would cost 30% less than administration by the intramammary route: 2.8 € versus 4.0 €, respectively (based on current retail prices of the products in Greece), which can also play a role in the selection by the prescribing veterinarians. However, when one also takes into account the bacteriological and, more importantly, the cytological results after treatment, the benefits of using the intramammary route become apparent.

In this respect, it should also be noted that, in cattle herds, the systematic administration of antibiotics for the treatment of mastitis was associated with a more frequent isolation of multi-drug resistant non-aureus staphylococci in comparison to using the intramammary products [21]. The longer period of staphylococcal shedding by ewes after treatment via the injectable route would increase the risk of infection of other animals in the flock. After the remission of clinical signs, farmers would start milking animals again, as the result of which bacteria could enter into the milking system and disseminate to other animals in the farm.

Moreover, the somatic cell counts would remain high for a longer period after treatment with an injectable form. That may contribute to increasing the somatic cell counts in the bulk tank milk, which can adversely affect the prices offered to farmers by dairy companies [22]. Furthermore, the earlier shift of the type of leucocytes, from neutrophils to lymphocytes, in the mammary secretion samples from the ewes in group T2 (D10 vs. D12–D13 in group T1) is an indicator that, in the former animals, the development of the chronic state started earlier. In contrast, in the latter animals, the defence process progressed and resulted in their earlier overall cure, as corroborated by the clinical, bacteriological, and cytological findings.

It is noteworthy that in an extensive study performed recently an association was found between the route of the administration of the drugs for the treatment of clinical mastitis and the somatic cell counts and total bacterial counts in the bulk tank milk of the farms: 0.393 × 10^6^ cells mL^−1^ and 309 × 10^3^ cfu mL^−1^, respectively, in farms where treatment was performed by the intramammary route, versus 0.743 × 10^6^ cells mL^−1^ and 1413 × 10^3^ cfu mL^−1^, respectively, in farms where treatment was performed by the injectable route [11]. The results of the current experimental study fully align with that evidence from the field.

### 3.3. Withdrawl Periods

Neither of the two pharmaceutical forms used in the present study has a specific licence for administration to sheep. Hence, in accordance with the regulations regarding veterinary drugs in the European Union, the prescribing veterinarians must define the withdrawal period, which for milk cannot be shorter than 7 days. The correct usage of antimicrobial agents, based on sound scientific principles and full compliance with the regulations and policies, is important for the improvement of the welfare of farm animals and also for minimizing the development of antibiotic resistance. This is another type of people–animal interaction that can possibly be considered as another approach within the ‘One Health’ concept.

Moreover, this lengthy withdrawal period can affect the prescribing preferences as this would mean a higher cost for farmers. This, however, does not decrease the value of the work as a comparative study between two routes of administration and two time points for the initiation of the treatment of mastitis in sheep.

## 4. Materials and Methods

### 4.1. Study Design

#### 4.1.1. Animals and Allocation into Groups

Multiparous animals of the Karagouniko-cross breed (*n* = 18 in total) were used in the study, which was performed in central Greece in 2013. Standard health management procedures (feeding, vaccinations, anthelmintic administration, etc.) were performed in all ewes throughout their pregnancy [23].

Ten days after lambing, the animals were allocated at random to three equal groups: T1, T2, or T3 (*n* = 6 each). At that stage, the bodyweight of the animals ranged from 52 to 59 kg. A randomization schedule in a 1:1:1 ratio was performed (author G.C.F.) for the allocation of ewes in each of the three groups. There was no difference in the number of lactation periods between the ewes in the three groups (median (min.–max.) lactation period for T1: 3 (3–4), for T2: 3 (2–4), and for T3: 3 (2–4); *p* = 0.73). The animals in each group were penned together. On the 10th day post-partum, the ewes in groups T1 or T2 were challenged with a *Staphylococcus simulans* isolate, which had been recovered previously from a case of mastitis in a ewe [13].

Identification of the isolate was confirmed by means of the ‘API-Staph SYSTEM’ (bioMérieux, Marcy-l’ Etoile, France) and a combination of biochemical tests [24]. No antibiotic susceptibility was detected in the challenge isolate; disks containing ampicillin (10 mg), cefoperazone (75 mg), cefuroxime (30 mg), clindamycin (2 mg), cloxacillin (1 mg), enrofloxacin (5 mg), erythromycin (15 mg), gentamicin (10 mg), methicillin (5 mg), neomycin (30 mg), penicillin-G (10 i.u.), and tetracycline (30 mg) were used [25].

Before and after intramammary inoculation, the animals were clinically examined in order to monitor their health status. Moreover, milk (from non-inoculated mammary glands) or mammary secretion (after inoculation) samples were collected to further monitor the animals, as detailed in Section 4.2.

#### 4.1.2. Inoculations

For inoculation, the challenge isolate was reconstituted into liquid and on solid media. Then, it was cultured on Columbia blood agar and confirmed for purity. The challenge isolate was inoculated into soy broth (BioMerieux, Marcy-l’-Étoile, France) and the liquid media were incubated in aerobic conditions at 37 °C for a period of 5 h. Then, the broth culture was serially diluted in phosphate-buffered saline (PBS), pH 7.3; finally, 0.2 mL of the desired dilution was drawn into a syringe. The inoculum contained in total 4.55 × 10^6^ to 4.80 × 10^5^ c.f.u., as measured by the method of Miles and Misra (or surface viable count), which is employed in bacteriology to count the number of colony-forming units (c.f.u.) in a bacterial suspension or homogenate [26].

For inoculation, the ewes were injected intramuscularly with oxytocin (dose: 10 IU) and their mammary glands were completely milked out. The mammary gland to be inoculated was chosen at random (at the toss of a coin: left or right gland). Inoculation was performed through the teat, directly into the *sinus lactiferous*. The challenge was performed under strict aseptic conditions. A sterile fine plastic catheter 20 G (Abbocath, Abbot) was inserted into the teat; a syringe containing the inoculum was attached to the catheter and the bacterial suspension was injected [27,28]. The same technique was used to inject a volume of 0.2 mL PBS into the contralateral mammary cistern of each ewe, as a control.

#### 4.1.3. Pharmaceutical Treatments

After the challenge, the ewes in groups T1 and T2 were treated by intramammary administration of a pharmaceutical product containing a combination of ampicillin (188.2 mg per tube) and dicloxacillin (191.4 mg per tube) in the form of infusion for intramammary treatment (Cloxalene Plus intramammary oint.; Fatro, Ozzano dell’Emilia, Italy). The licensed schedule of the product was followed, and two administrations of the product (one tube on each occasion) were given to each ewe, with a 12-h interval between them. The teat was cleaned by using single-use wipes with disinfectant solution; then, the tube was inserted into the teat and the product was infused into the *sinus lactiferous*; thereafter, the mammary gland was gently massaged for approximately 30 s.

The ewes in group T3 were treated by intramuscular injection of a pharmaceutical product containing a combination of ampicillin (95.3 mg per mL) and dicloxacillin (47.8 mg per mL) in the form of an injectable suspension (Cloxalene Plus inj. susp.; Fatro). The licensed schedule of the product was followed, and three injections of the product were given to each ewe, with 24-h intervals; the dose rate was 0.75 mL of the product per 10 kg bodyweight.

In the ewes in groups T1 and T3, treatment started immediately when clinical signs of mastitis were first detected during the periodic examination of the ewes. In the ewes in group T2, treatment started 24 h after clinical signs of mastitis were first detected. Summaries of the treatment protocols are in Table 4.

### 4.2. Examination of Animals and Collections of Samples

After lambing, general clinical examinations of all the experimental ewes were routinely carried out at 2-day intervals [29]. The rectal temperature was recorded. Before inoculation, on days D–4 and D–1, a standardized detailed clinical examination of the udder (observation, palpation, comparison between glands) was performed (author N.G.C.V.), as described before [30].

After challenge, the animals were monitored and examined at 6-h intervals until administration of treatment. After treatment (D0), detailed clinical examinations were performed twice daily up to D7 (14 examination points), then daily up to D14 (7 examination points), and thereafter every three days up to D41 (9 examination points).

During the clinical examinations, the clinical severity of the disease was evaluated by assessing the following five different mammary clinical signs, which were observed and recorded separately: appearance of milk (or mammary secretion), temperature of mammary gland, presence of pain in the detection gland, oedema of the detection gland, size of the detection gland [31].

For evaluation of the clinical severity of the infection, five different mammary clinical signs were monitored and were each scored on a 0 to 4 scale, according to their severity (Table 5) [31]. The total score for clinical severity of mastitis was produced by summing the individual scores given for each clinical sign. Thus, the total score for clinical severity in an individual animal at any examination time point could range from 0 to 20.

On each examination time point, mammary secretion samples were collected from both mammary glands of all animals, under strict aseptic conditions. In total, 5 to 10 mL of mammary secretion was collected into a sterile container. Separate samples were collected from each mammary gland [30,31].

### 4.3. Laboratory Examinations

Milk or mammary secretion samples (10 μL) were cultured using Columbia sheep blood agar plates incubated aerobically at 37 °C for 48 h. If no growth was observed, the plates were incubated for another 24 h. Bacterial colonies morphologically similar to those of *S. simulans* (cream white, circular, convex, smooth, uniform, butyrous, shiny) present in pure culture were considered to be those of the challenge organism. A proportion of the isolates thus recovered (20%, i.e., 20/98 of isolates recovered from challenge to initiation of treatment and 46/229 of isolates recovered after initiation of treatment), randomly selected by use of an electronic random number generator (www.randomresult.com (accessed on 5 October 2022)), were fully identified by means of the API Staph quick biochemical identification strips (bioMérieux).

Milk or mammary secretion samples from glands without clinical signs were assessed for cell counting using the Microscopic cell counting method (IDF reference method) [32]. Moreover, smears were made from all mammary secretion samples; these were dried and stained using the Giemsa method for evaluation of the types of leucocyte subpopulations.

### 4.4. Data Management and Analysis

For confirmation of the identity of the staphylococcal isolates obtained after inoculation, a threshold of >85% was employed in the API test, as suggested by Renneberg et al. [33].

For all statistical analyses, somatic cell counts (SCC) were transformed to somatic cell scores (SCS), as described by Wiggans and Shook [34] and Franzoi et al. [35]: SCS = log_2_(SCC/100) + 3. Then, for presentation of the results, the transformed findings were back-transformed into 100 × 2^(SCS−3)^.

Complete cure of an inoculated mammary gland was defined when (a) the absence of clinical signs in the inoculated mammary gland, (b) no recovery of *Staphylococcus* sp. from mammary secretion from the inoculated gland, and (c) somatic cell counts below 0.50 × 10^6^ or 0.75 × 10^6^ or 1.00 × 10^6^ cells mL^−1^ in mammary secretion from the inoculated gland were seen concurrently in an experimental ewe.

If an event of interest (presence of clinical signs, isolation of staphylococci from the mammary secretion, high somatic cell counts (above each of the three predefined thresholds), or complete cure) was noted on an examination point but not on the following one, it was deemed that the event was resolved halfway between the two examination points. If an event of interest was noted intermittently, it was deemed that it had been resolved completely after the examination point in which it was recorded for the last time. Based on the above, it was possible to calculate an estimate of the duration that the events of interest occurred in each experimental ewe.

Differences in clinical scores between groups were compared by using analysis of covariance. The Wilcoxon 1-sample test was employed to test for differences in clinical scores between the inoculated and the contralateral mammary gland of each ewe. Differences in proportions were compared by using Pearson’s chi-square test. Differences in somatic cell counts between groups were compared by using analysis of variance. Differences in the duration of the occurrence of the various events of interest were compared between groups by means of the Kruskal–Wallis test.

In all cases, significance was defined at *p* < 0.05.

## 5. Conclusions

The findings provide further information regarding the treatment of clinical mastitis in ewes. The early initiation of treatment resulted in a significantly improved cure of the infection, as corroborated by the results of the clinical, bacteriological, and cytological examinations. When comparing the intramammary and injectable routes of antibiotic administration, there was some benefit of the former, primarily in the post-treatment somatic cell counts, which can be reflected in the quality of milk produced at the farm.

## Figures and Tables

**Table 1:** Scores (median (interquartile range)) for clinical severity of mastitis in three groups of ewes after intramammary treatment performed immediately (T1) or with a 24 h delay (T2) or after systemic treatment performed immediately (T3).

Day after Treatment ^1^	Group T1	Group T2	Group T3
D0	5.5 (2.0)	8.0 (2.5)	6.0 (1.5)
D0 + 12 h	6.0 (1.5)	9.5 (2.5)	6.5 (1.0)
D1	6.0 (1.0)	9.5 (2.0)	7.0 (1.5)
D1 + 12 h	6.0 (2.0)	8.5 (1.0)	7.0 (1.0)
D2	3.5 (2.0)	8.5 (1.0)	5.0 (1.0)
D2 + 12 h	3.0 (1.0)	6.0 (1.5)	4.0 (1.0)
D3	2.5 (1.0)	4.5 (1.0)	3.0 (0.5)
D3 + 12 h	1.5 (1.0)	3.0 (0.5)	2.0 (1.0)
D4	1.0 (0.5)	3.0 (0.5)	1.0 (1.0)
D4 + 12 h	1.0 (1.0)	2.5 (0.5)	1.0 (0.0)
D5	0.0 (1.0)	2.0 (1.0)	0.0 (1.0)
D5 + 12 h	0.0 (1.0)	1.5 (1.0)	0.0 (1.0)
D6	0.0 (1.0)	1.0 (0.0)	0.0 (1.0)
D6 + 12 h	0.0 (0.5)	1.0 (0.0)	0.0 (1.0)
D7	0.0 (0.0)	0.5 (1.0)	0.0 (0.5)
D8	0.0 (0.0)	0.0 (1.0)	0.0 (0.5)
D9	0.0 (0.0)	0.0 (1.0)	0.0 (0.5)
D10	0.0 (0.0)	0.0 (0.5)	0.0 (0.0)
D11	0.0 (0.0)	0.0 (0.5)	0.0 (0.0)
D12	0.0 (0.0)	0.0 (0.5)	0.0 (0.0)
D13	0.0 (0.0)	0.0 (0.5)	0.0 (0.0)
D14	0.0 (0.0)	0.0 (0.5)	0.0 (0.0)
D17	0.0 (0.0)	0.0 (0.5)	0.0 (0.0)
D20	0.0 (0.0)	0.0 (0.5)	0.0 (0.0)
D23-D41	0.0 (0.0)	0.0 (0.0)	0.0 (0.0)

^1^ D0 + 12 h to D6 + 12 h, *p* < 0.04 for differences between groups on each sampling point; thereafter, *p* > 0.33 on each sampling point.

**Table 2:** Proportion of mammary secretion samples from which staphylococci with colonial morphology similar to that of the challenge organism were recovered, in three groups of ewes after intramammary treatment performed immediately (T1) or with a 24 h delay (T2) or after systemic treatment performed immediately (T3).

Bacterial Recovery	Group T1	Group T2	Group T3	*p*
D0 + 12 h to D6 + 12 h	50/78 (64.1%)	74/78 (94.9%)	75/78 (96.2%)	<0.0001
D7 to D41	1/102 (1.0%)	17/102 (16.7%)	12/102 (11.8%)	0.005

**Table 3:** Somatic cell counts (× 10^6^ cells mL^−1^) in mammary secretion samples from ewes in three groups after intramammary treatment performed immediately (T1) or with a 24 h delay (T2) or after systemic treatment performed immediately (T3) (mean, 95% confidence interval of the mean).

Somatic Cell Counts	Group T1	Group T2	Group T3	*p*
D0 + 12 h to D6 + 12 h	1.923 (1.732–2.132)	n/a	2.222 (2.045–2.415)	0.039
D7 to D14	1.408 (1.312–1.518)	2.188 (1.935–2.466)	1.699 (1.561–1.843)	<0.0001
D17 to D41	0.470 (0.430–0.515)	0.783 (0.698–0.878)	0.578 (0.518–0.643)	<0.0001
Entire study period	0.895 (0.791–0.836)	1.192 (1.044–1.368)	1.063 (0.941–1.199)	0.008

**Table 4:** Summaries of treatment protocols in three groups of ewes after intramammary challenge with *S. simulans* on the 10th day after lambing.

Group	Pharmaceutical Products Administered	Dose	Route	Initiation of Administration of Treatment
T1 (*n* = 6)	Ampicillin and dicloxacillin	1 tube ×2, every 12 h	Intramammary	With onset of clinical signs
T2 (*n* = 6)	Ampicillin and dicloxacillin	1 tube ×2, every 12 h	Intramammary	24 h after onset of clinical signs
T3 (*n* = 6)	Ampicillin and dicloxacillin	0.75 mL per 10 kg bw, ×3, every 24 h	Systemic	With onset of clinical signs

**Table 5:** Description of scores for mammary clinical signs observed and recorded during the clinical examination [6].

Score	Mammary Secretion	Temperature	Pain	Oedema	Size
0	normal (milk)	normal	none	none	normal
1	flakes	warm +	slight	slight	10–35% increase
2	clots	warm + +	moderate	moderate	36–65% increase
3	serous	warm + + +	marked	marked	66–100% increase
4	blood	cold	severe	widespread	decrease

## Data Availability

All relevant data are included as Supplementary Materials.

## Supplementary Materials

The following supporting information can be downloaded at: https://www.mdpi.com/article/10.3390/pathogens11101164/s1, Table S1: detailed results of total scores for clinical severity of mastitis in three groups of ewes after intramammary treatment performed immediately (T1) or with a 24 h delay (T2) or after systemic treatment performed immediately (T3) throughout the study, Table S2: detailed results of bacteriological examination and recovery of *Staphylococcus* sp. in three groups of ewes after intramammary treatment performed immediately (T1) or with a 24 h delay (T2) or after systemic treatment performed immediately (T3) throughout the study, Table S3: scores for clinical severity of mastitis in accord with bacterial recovery from mammary secretion samples in three groups of ewes after intramammary treatment performed immediately (T1) or with a 24 h delay (T2) or after systemic treatment performed immediately (T3) (mean ± standard error of the mean), Table S4: detailed results of somatic cell counts in three groups of ewes after intramammary treatment performed immediately (T1) or with a 24 h delay (T2) or after systemic treatment performed immediately (T3) throughout the study, Table S5: proportion of mammary secretion samples with high somatic cell counts (i.e., above three different thresholds) in three groups of ewes after intramammary treatment performed immediately (T1) or with a 24 h delay (T2) or after systemic treatment performed immediately (T3), Table S6: duration (median (minimum–maximum)) of high somatic cell counts (i.e., above three different thresholds) in mammary secretion samples in three groups of ewes after intramammary treatment performed immediately (T1) or with a 24 h delay (T2) or after systemic treatment performed immediately (T3), Table S7: median day of achievement of complete cure of mastitis in three groups of ewes after intramammary treatment performed im-mediately (T1) or with a 24 h delay (T2) or after systemic treatment performed immediately (T3), according to the threshold for normality used in somatic cell counting.
