# Supplementary material for: Comparison of the Efficacy of Intramammary or Injectable Antibiotic Administration against Staphylococcal Mastitis in Ewes
Source: Pathogens. 2022 Oct 9;11(10):1164. doi: 10.3390/pathogens11101164 (PMC9607280; doi:10.3390/pathogens11101164)
Supplement: Supplementary file 1 [file pathogens-11-01164-s001.zip › pathogens-1941350-supplementary.pdf]

Comparison of the Efficacy of Intramammary or Injectable Antibiotic Administration Against Staphylococcal Mastitis in Ewes

Natalia G.C. Vasileiou, George C. Fthenakis and Vasia S. Mavrogianni

Table S1. Detailed results of total scores for clinical severity of mastitis in three groups of ewes after intramammary treatment performed immediately (T1) or with a 24 h delay (T2) or after systemic treatment performed immediately (T3) throughout the study.

Day after initiation of treatment	Animal no. within each group	Group T1	Group T2	Group T3
D0	1	6	6	4
	2	5	6	5
	3	4	9	6
	4	6	8	6
	5	6	8	6
	6	4	8	6
	Group median score	5.5	8	6
D0 + 12 h	1	7	8	6
	2	6	8	6
	3	6	10	7
	4	7	11	7
	5	6	10	6
	6	5	9	7
	Group median score	6	9.5	6.5
D1	1	6	10	7
	2	6	8	6
	3	6	9	8
	4	6	11	8
	5	7	10	7
	6	7	9	7
	Group median score	6	9.5	7
D1 + 12 h	1	6	9	7
	2	6	9	6
	3	6	8	7
	4	5	9	7
	5	7	8	6
	6	4	8	7
	Group median score	6	8.5	7
D2	1	4	9	6
	2	4	9	5
	3	2	8	5
	4	3	9	6
	5	4	8	5
	6	2	8	5
	Group median score	3.5	8.5	5

D2 + 12 h	1	3	6	4
	2	3	6	4
	3	2	5	3
	4	3	6	3
	5	3	7	4
	6	2	7	4
	Group median score	3	6	4
D3	1	3	5	3
	2	2	5	3
	3	2	4	3
	4	3	5	2
	5	3	4	3
	6	2	4	3
	Group median score	2.5	4.5	3
D3 + 12 h	1	2	3	2
	2	1	3	2
	3	2	3	2
	4	1	2	2
	5	1	3	1
	6	2	3	1
	Group median score	1.5	3	2
D4	1	1	2.5	1
	2	1	3	2
	3	1	3	2
	4	1	2	1
	5	1	2.5	1
	6	2	3	1
	Group median score	1	2.75	1
D4 + 12 h	1	1	2	1
	2	1	3	1
	3	1	3	1
	4	1	2	1
	5	2	2	1
	6	2	3	1
	Group median score	1	2	1
D5	1	0	2	1
	2	1	2	0
	3	0	2	0
	4	0	1	0
	5	0	2	0
	6	1	1	1
	Group median score	0	2	0
D5 + 12 h	1	0	1	1
	2	1	2	0
	3	0	1	0
	4	0	2	0
	5	0	2	0
	6	1	1	1
	Group median score	0	1.5	0

D6	1	0	1	1
	2	1	1	0
	3	0	1	0
	4	0	1	0
	5	1	1	0
	6	0	1	1
	Group median score	0	1	1
D6 + 12 h	1	0	1	1
	2	1	1	0
	3	0	1	0
	4	0	1	0
	5	0	1	0
	6	0	1	1
	Group median score	0	1	0
D7	1	0	0	1
	2	0	1	0
	3	0	0	0
	4	0	1	0
	5	0	1	0
	6	0	0	0
	Group median score	0	0.5	0
D8	1	0	0	1
	2	0	1	0
	3	0	0	0
	4	0	0	0
	5	0	1	0
	6	0	0	0
	Group median score	0	0	0
D9	1	0	0	1
	2	0	1	0
	3	0	0	0
	4	0	0	0
	5	0	1	0
	6	0	0	0
	Group median score	0	0	0
D10	1	0	0	0
	2	0	0	0
	3	0	0	0
	4	0	0	0
	5	0	1	0
	6	0	0	0
	Group median score	0	0	0
D11	1	0	0	0
	2	0	0	0
	3	0	0	0
	4	0	0	0
	5	0	1	0
	6	0	0	0
	Group median score	0	0	0

D12	1	0	0	0
	2	0	0	0
	3	0	0	0
	4	0	0	0
	5	0	1	0
	6	0	0	0
	Group median score	0	0	0
D13	1	0	0	0
	2	0	0	0
	3	0	0	0
	4	0	0	0
	5	0	1	0
	6	0	0	0
	Group median score	0	0	0
D14	1	0	0	0
	2	0	0	0
	3	0	0	0
	4	0	0	0
	5	0	1	0
	6	0	0	0
	Group median score	0	0	0
D17	1	0	0	0
	2	0	0	0
	3	0	0	0
	4	0	0	0
	5	0	1	0
	6	0	0	0
	Group median score	0	0	0
D20	1	0	0	0
	2	0	0	0
	3	0	0	0
	4	0	0	0
	5	0	1	0
	6	0	0	0
	Group median score	0	0	0
D23	1	0	0	0
	2	0	0	0
	3	0	0	0
	4	0	0	0
	5	0	0	0
	6	0	0	0
	Group median score	0	0	0
D26	1	0	0	0
	2	0	0	0
	3	0	0	0
	4	0	0	0
	5	0	0	0
	6	0	0	0
	Group median score	0	0	0

D29	1	0	0	0
	2	0	0	0
	3	0	0	0
	4	0	0	0
	5	0	0	0
	6	0	0	0
	Group median score	0	0	0
D32	1	0	0	0
	2	0	0	0
	3	0	0	0
	4	0	0	0
	5	0	0	0
	6	0	0	0
	Group median score	0	0	0
D35	1	0	0	0
	2	0	0	0
	3	0	0	0
	4	0	0	0
	5	0	0	0
	6	0	0	0
	Group median score	0	0	0
D38	1	0	0	0
	2	0	0	0
	3	0	0	0
	4	0	0	0
	5	0	0	0
	6	0	0	0
	Group median score	0	0	0
D41	1	0	0	0
	2	0	0	0
	3	0	0	0
	4	0	0	0
	5	0	0	0
	6	0	0	0
	Group median score	0	0	0

Table S2. Detailed results of bacteriological examination and recovery of staphyloocci (+: recovery, -: no recovery) from three groups of ewes after intramammary treatment performed immediately (T1) or with a 24 h delay (T2) or after systemic treatment performed immediately (T3) throughout the study.

Day after initiation of treatment	Animal no. within each group	Group T1	Group T2	Group T3
D0	1	+	+	+
	2	+	+	+
	3	+	+	+
	4	+	+	+
	5	+	+	+
	6	+	+	+
	No. positives	6	6	6
D0 + 12 h	1	+	+	+
	2	+	+	+
	3	+	+	+
	4	+	+	+
	5	+	+	+
	6	+	+	+
	No. positives	6	6	6
D1	1	+	+	+
	2	+	+	+
	3	+	+	+
	4	+	+	+
	5	+	+	+
	6	+	+	+
	No. positives	6	6	6
D1 + 12 h	1	+	+	+
	2	+	+	+
	3	+	+	+
	4	+	+	+
	5	+	+	+
	6	+	+	+
	No. positives	6	6	6
D2	1	+	+	+
	2	+	+	+
	3	+	+	+
	4	+	+	+
	5	+	+	+
	6	+	+	+
	No. positives	6	6	6
D2 + 12 h	1	+	+	+
	2	+	+	+
	3	+	+	+
	4	+	+	+
	5	+	+	+
	6	+	+	+
	No. positives	6	6	6

D3	1	+	+	+
	2	+	+	+
	3	+	+	+
	4	+	+	+
	5	+	+	+
	6	+	+	+
	No. positives	6	6	6
D3 + 12 h	1	-	+	+
	2	+	+	+
	3	-	+	+
	4	+	+	+
	5	-	+	+
	6	+	+	+
	No. positives	3	6	6
D4	1	-	+	-
	2	+	+	+
	3	-	+	+
	4	+	+	+
	5	-	+	+
	6	-	+	+
	No. positives	2	6	5
D4 + 12 h	1	-	+	-
	2	-	+	+
	3	-	+	+
	4	+	+	+
	5	-	+	+
	6	-	+	+
	No. positives	1	6	5
D5	1	-	+	+
	2	+	+	+
	3	-	+	+
	4	+	+	+
	5	-	+	+
	6	-	+	+
	No. positives	2	6	3
D5 + 12 h	1	-	+	+
	2	+	+	+
	3	-	-	+
	4	+	+	+
	5	-	+	+
	6	-	+	+
	No. positives	2	5	6
D6	1	-	+	+
	2	+	+	+
	3	-	+	+
	4	+	+	+
	5	-	-	+
	6	-	+	+
	No. positives	2	3	2

D6 + 12 h	1	-	+	+
	2	+	+	-
	3	-	-	+
	4	+	+	+
	5	-	-	+
	6	-	+	+
	No. positives	2	3	2
D7	1	-	+	+
	2	-	-	-
	3	-	-	+
	4	-	+	+
	5	-	+	+
	6	-	+	-
	No. positives	1	2	1
D8	1	-	-	+
	2	+	-	-
	3	-	-	-
	4	-	+	-
	5	-	+	+
	6	-	+	+
	No. positives	0	2	2
D9	1	-	-	+
	2	-	-	-
	3	-	-	-
	4	-	+	-
	5	-	+	-
	6	-	-	+
	No. positives	1	2	2
D10	1	-	-	-
	2	-	-	-
	3	-	-	-
	4	-	+	-
	5	-	+	-
	6	-	-	-
	No. positives	1	2	0
D11	1	-	-	-
	2	-	-	-
	3	-	-	-
	4	-	-	-
	5	-	+	-
	6	-	-	-
	No. positives	0	1	0
D12	1	-	-	+
	2	-	-	-
	3	-	-	-
	4	-	-	-
	5	-	+	-
	6	-	-	-
	No. positives	0	1	1

D13	1	-	-	+
	2	-	-	-
	3	-	-	-
	4	-	-	-
	5	-	+	-
	6	-	-	-
	No. positives	0	1	1
D14	1	-	-	+
	2	-	-	-
	3	-	-	-
	4	-	-	-
	5	-	+	-
	6	-	-	-
	No. positives	0	1	1
D17	1	-	-	-
	2	-	-	-
	3	-	-	-
	4	-	-	-
	5	-	+	-
	6	-	-	-
	No. positives	0	1	0
D20	1	-	-	-
	2	-	-	-
	3	-	-	-
	4	-	-	-
	5	-	-	-
	6	-	-	-
	No. positives	0	0	0
D23	1	-	-	-
	2	-	-	-
	3	-	-	-
	4	-	-	-
	5	-	-	-
	6	-	-	-
	No. positives	0	0	0
D26	1	-	-	-
	2	-	-	-
	3	-	-	-
	4	-	-	-
	5	-	-	-
	6	-	-	-
	No. positives	0	0	0
D29	1	-	-	-
	2	-	-	-
	3	-	-	-
	4	-	-	-
	5	-	-	-
	6	-	-	-
	No. positives	0	0	0

D32	1	-	-	-
	2	-	-	-
	3	-	-	-
	4	-	-	-
	5	-	+	-
	6	-	-	-
	No. positives	0	0	0
D35	1	-	-	-
	2	-	-	-
	3	-	-	-
	4	-	-	-
	5	-	-	-
	6	-	-	-
	No. positives	0	0	0
D38	1	-	-	-
	2	-	-	-
	3	-	-	-
	4	-	-	-
	5	-	-	-
	6	-	-	-
	No. positives	0	0	0
D41	1	-	-	-
	2	-	-	-
	3	-	-	-
	4	-	-	-
	5	-	-	-
	6	-	-	-
	No. positives	0	0	0

Table S3. Scores for clinical severity of mastitis in accord with bacterial recovery from mammary secretion samples in three groups of ewes after intramammary treatment performed immediately (T1) or with a 24 h delay (T2) or after systemic treatment performed immediately (T3) (mean ± standard error of the mean).

Bacterial recovery	Group T1	Group T2	Group T3	*p*
No	0.16 ± 0.04	0.09 ± 0.03	0.02 ± 0.02	0.017
Yes	3.50 ± 0.30	4.22 ± 0.34	2.78 ± 0.27	0.003

Table S4. Detailed results of somatic cell counts (× 10^3 cells ml^{-1}) in three groups of ewes after intramammary treatment performed immediately (T1) or with a 24 h delay (T2) or after systemic treatment performed immediately (T3) throughout the study.

Day after initiation of treatment	Animal no. within each group	Group T1	Group T2	Group T3
D0	1	n/a	n/a	n/a
	2	n/a	n/a	n/a
	3	n/a	n/a	n/a
	4	n/a	n/a	n/a
	5	n/a	n/a	n/a
	6	n/a	n/a	n/a
D0 + 12 h	1	n/a	n/a	n/a
	2	n/a	n/a	n/a
	3	n/a	n/a	n/a
	4	n/a	n/a	n/a
	5	n/a	n/a	n/a
	6	n/a	n/a	n/a
D1	1	n/a	n/a	n/a
	2	n/a	n/a	n/a
	3	n/a	n/a	n/a
	4	n/a	n/a	n/a
	5	n/a	n/a	n/a
	6	n/a	n/a	n/a
D1 + 12 h	1	n/a	n/a	n/a
	2	n/a	n/a	n/a
	3	n/a	n/a	n/a
	4	n/a	n/a	n/a
	5	n/a	n/a	n/a
	6	n/a	n/a	n/a
D2	1	n/a	n/a	n/a
	2	n/a	n/a	n/a
	3	n/a	n/a	n/a
	4	n/a	n/a	n/a
	5	n/a	n/a	n/a
	6	n/a	n/a	n/a
D2 + 12 h	1	n/a	n/a	n/a
	2	n/a	n/a	n/a
	3	n/a	n/a	n/a
	4	n/a	n/a	n/a
	5	n/a	n/a	n/a
	6	n/a	n/a	n/a
D3	1	n/a	n/a	n/a
	2	n/a	n/a	n/a
	3	n/a	n/a	n/a
	4	n/a	n/a	n/a
	5	n/a	n/a	n/a
	6	n/a	n/a	n/a

D3 + 12 h	1	n/a	n/a	n/a
	2	n/a	n/a	n/a
	3	n/a	n/a	n/a
	4	n/a	n/a	n/a
	5	n/a	n/a	n/a
	6	n/a	n/a	n/a
D4	1	n/a	n/a	n/a
	2	n/a	n/a	n/a
	3	n/a	n/a	n/a
	4	n/a	n/a	n/a
	5	n/a	n/a	n/a
	6	n/a	n/a	n/a
D4 + 12 h	1	n/a	n/a	n/a
	2	n/a	n/a	n/a
	3	n/a	n/a	n/a
	4	n/a	n/a	n/a
	5	n/a	n/a	n/a
	6	n/a	n/a	n/a
D5	1	1420	n/a	n/a
	2	n/a	n/a	2480
	3	2070	n/a	1590
	4	2450	n/a	2720
	5	1880	n/a	2550
	6	n/a	n/a	n/a
D5 + 12 h	1	1450	n/a	n/a
	2	n/a	n/a	2350
	3	2100	n/a	1740
	4	2400	n/a	2600
	5	2020	n/a	2630
	6	n/a	n/a	n/a
D6	1	1400	n/a	n/a
	2	n/a	n/a	1940
	3	2070	n/a	1960
	4	2460	n/a	2500
	5	2150	n/a	2480
	6	n/a	n/a	n/a
D6 + 12 h	1	1280	n/a	n/a
	2	n/a	n/a	1850
	3	1960	n/a	2010
	4	2520	n/a	2270
	5	2060	n/a	2320
	6	1690	n/a	n/a
D7	1	1470	1360	n/a
	2	1190	n/a	2280
	3	1670	3490	1920
	4	2630	n/a	2670
	5	2430	n/a	1940
	6	1730	2980	2110

D8	1	1280	1680	n/a
	2	1420	n/a	2160
	3	2120	3800	1950
	4	1930	3870	2610
	5	1840	n/a	1750
	6	1780	2620	1820
D9	1	1250	1540	n/a
	2	1470	n/a	1670
	3	1480	3860	1880
	4	1550	2380	2470
	5	1350	n/a	2050
	6	1790	2550	1680
D10	1	1620	1690	1970
	2	1260	2340	1370
	3	1390	2960	1720
	4	1830	3780	2650
	5	1530	n/a	2560
	6	1940	1850	2070
D11	1	1440	1970	1700
	2	1290	2980	1560
	3	1340	2230	1760
	4	1590	4870	2710
	5	1330	n/a	2370
	6	1780	1880	1970
D12	1	1550	1650	1690
	2	1860	2140	1290
	3	1180	2990	1240
	4	950	2730	1950
	5	1130	n/a	1350
	6	980	1970	1820
D13	1	1120	1900	990
	2	1660	1860	1180
	3	1230	2480	1090
	4	1260	1650	1670
	5	970	n/a	1250
	6	1020	1420	1450
D14	1	920	1220	870
	2	1050	1480	1070
	3	960	1440	1060
	4	940	1880	1270
	5	920	n/a	1540
	6	1420	940	1380
D17	1	780	1320	980
	2	530	1450	840
	3	880	1190	930
	4	920	1400	770
	5	1030	n/a	1270
	6	930	830	1620

D20	1	580	1070	800
	2	520	1330	800
	3	680	1010	760
	4	830	1270	350
	5	770	n/a	770
	6	920	930	600
D23	1	400	920	950
	2	370	880	940
	3	590	1320	840
	4	760	1490	710
	5	570	1550	910
	6	620	860	490
D26	1	330	780	710
	2	310	990	550
	3	580	970	480
	4	810	1270	870
	5	370	1470	860
	6	610	770	440
D29	1	560	690	690
	2	470	690	650
	3	470	780	410
	4	650	840	780
	5	470	930	600
	6	720	760	450
D32	1	470	740	570
	2	310	540	580
	3	450	980	470
	4	480	880	640
	5	270	1060	570
	6	540	670	520
D35	1	340	470	320
	2	480	360	540
	3	370	640	450
	4	470	760	540
	5	260	780	290
	6	330	610	390
D38	1	210	540	260
	2	290	420	260
	3	280	590	680
	4	400	590	480
	5	240	670	420
	6	390	550	450
D41	1	270	440	360
	2	290	280	370
	3	360	580	570
	4	280	410	380
	5	350	390	310
	6	360	410	490

Table S5. Proportion of mammary secretion samples with high somatic cell counts (i.e., above three different thresholds) in three groups of ewes after intramammary treatment performed immediately (T1) or with a 24 h delay (T2) or after systemic treatment performed immediately (T3).

Threshold for somatic cell counts (cells mL^{-1})	Group T1	Group T2	Group T3	*p*
0.50×10^6	89 / 119	80 / 88	98 / 115	0.007
0.75×10^6	75 / 119	67 / 88	78 / 115	0.13
1.00×10^6	61 / 119	49 / 88	66 / 115	0.63

Table S6. Duration (median (minimum-maximum)) of high somatic cell counts (i.e., above three different thresholds) in mammary secretion samples in three groups of ewes after intramammary treatment performed immediately (T1) or with a 24 h delay (T2) or after systemic treatment performed immediately (T3).

Threshold for somatic cell counts (cells mL^{-1})	Group T1	Group T2	Group T3	*p*
0.50×10^6	29.0 d (21.5 – 33.5 d)	39.5 d (33.5 – 41.0 d)	35.0 d (33.5 – 41.0 d)	0.004
0.75×10^6	20.0 d (15.5 – 27.5 d)	32.0 d (27.5 – 36.5 d)	24.5 d (18.5 – 27.5 d)	0.004
1.00×10^6	14.5 d (13.5 – 18.5 d)	23.0 d (13.5 – 33.5 d)	15.5 d (12.5 – 18.5 d)	0.039

Table S7. Median day of achievement of complete cure of mastitis in three groups of ewes after intra-mammary treatment performed immediately (T1) or with a 24 h delay (T2) or after systemic treatment performed immediately (T3), according to the threshold for normality used in somatic cell counting.

Threshold for somatic cell counts (cells mL^{-1})	Group T1	Group T2	Group T3	*p*
0.50×10^6	29.0 d	39.5 d	35.0 d	0.004
0.75×10^6	20.0 d	32.0 d	24.5 d	0.004
1.00×10^6	14.5 d	23.0 d	15.5 d	0.033
